# Occupational Health Knowledge and Safety Practice Among Hydropower Construction Workers in Nepal: A Cross‐Sectional Study

**DOI:** 10.1002/hsr2.71561

**Published:** 2025-11-23

**Authors:** Khadga Bahadur Shrestha, Swastika Gairhe, Amrit Bist, Mamata Rawal

**Affiliations:** ^1^ Manmohan Memorial Institute of Health Sciences Kathmandu Nepal; ^2^ Health Office Dadeldhura Sudurpaschim Province Nepal

**Keywords:** construction worker, knowledge, Nepal, occupational health hazards, safety practices

## Abstract

**Background and Aims:**

The construction sector in Nepal has expanded considerably, accompanied by various occupational health risks due to inadequate knowledge and safety protocols. This study evaluated the knowledge and safety practices of Construction workers working at a hydropower construction site.

**Methods:**

A cross‐sectional study was carried out at the Tanahun hydropower construction site in Tanahun among 316 participants using simple random sampling. Data was collected through face‐to‐face interview using the semi structure questionnaire. The descriptive and inferential statistics were performed through SPSS version 20. Binary logistic regression was used to assess the association between variables, where variables with variance inflation factor less than 2 were included in multivariable analysis. The variables with p‐values < 0.05 considered statistically significant at 95% confidence interval.

**Results:**

The study showed that 65% and 64% of the participants exhibited adequate knowledge and good practice of safety protocols, respectively. The prevalence of various health issues such as skin infections (27.8%), musculoskeletal problems (21.5%) and electric shocks (14.6%), were notably high among workers. Adequate knowledge was significantly correlated with a higher level of educational attainment (AOR = 4.73, 95% CI, 1.78–12.41), and an absence of a history of musculoskeletal disorders (AOR = 9.59, 95%, CI, 3.49–26.35). Furthermore, adequate knowledge was related to extended working hours (AOR = 2.95, 95% CI, 1.28–6.81) whereas good practices were related to reduced working hours (AOR = 1.86, 95% CI, 1.63–3.41) and married marital status (AOR = 16.41,95%, CI, 4.27–62.99).

**Conclusion:**

The study reveals that only two‐thirds of participants in hydropower construction have adequate knowledge and safety practices, emphasizing the need for special attention to employees with musculoskeletal issues, longer shifts and lower education to safeguard the health of workforce.

## 1 Introduction

The World Health Organization (WHO) emphasizes occupational health as a crucial aspect of workplace safety, aiming to prevent and manage work‐related illnesses and accidents that could harm employees' physical and mental health [1]. The construction industry is especially concerning due to risk of physical, chemical, ergonomic, organizational hazards [2]. The International Labor Organization (ILO) estimates that approximately 2.3 million individuals, both male and female, worldwide experience fatalities resulting from work related incidents or illnesses annually. This statistic translates to a staggering 6000 deaths occurring each day. On a global scale, there are approximately 340 million occurrences of occupational accidents and 160 million individuals affected by work‐related diseases annually [3]. Study showed that construction workers face a three to six times higher risk of accidents than other workers, and at least 108,000 workers die on the job site each year [4].

The construction sector in Latin America and the Caribbean was a major contributor to occupational incidents, including falls, injuries, and exposure to hazardous materials [5]. Even though 62% of Nigerian construction workers are well‐informed about workplace hazards, 86% of them disregard safety precautions leading to common health issues like skin rashes, muscle pain, and back pain [6]. Similarly, physical hazards like noise and chemical hazards such as paints were found prevalent on construction sites [7]. A comprehensive review conducted in South Asia found that poor working conditions, insufficient training, and a lack of knowledge are among the occupational health and safety issues that construction workers confront [8]. This region has a startlingly high rate of work‐related fatalities; in Bangladesh, for instance, 57% of construction workers report a history of injuries, while 87% lack health and safety training [9]. While 70% of construction workers in India's Kashmir Valley reported experiencing at least one sickness, with skin conditions being the most prevalent, hydropower construction accidents are widespread in Bhutan. The frequency of accidents in hydropower plant constructions was 58.7%, and 8.3% in other construction activities (e.g. residential, road, and bridge constructions). Similarly it also shows that only 51.5% indicated they were aware of the prevention of hazards, only 43.8% of workers were aware of safety representatives at their workplace and 61.1% of workers had undergone either an orientation program or some training on health and safety [10, 11].

A similar scenario is evident in Nepal. Even though the construction sector employs over a million people, accounts for 10%–11% of Nepal's (Gross Domestic Product) GDP, and uses 35% of the government budget [12]. Occupational safety and health (OSH) conditions are inadequate [13, 14]. According to studies conducted in Nepal, 18% of construction workers have accidents every year, and 62% of them lack information about occupational safety [1]. According to national OSH profile for Nepal Occupational accidents in 2019/20 were 53. Among them 38 were minor accidents, 7 major accidents, 8 fatal accidents [15]. The Nepal Labor Act (2017), which prohibits discrimination in compensation, stipulates that workers must work no more than 8 h per day or 48 h per week, and calls for sufficient welfare facilities and first aid kits, is frequently not carried out effectively [16]. According to research conducted in 2022, working conditions on building sites regularly defy legislative requirements [17].

The good OSH contributes to the establishment of a safe and healthy workplace, which is strongly linked to the many Sustainable Development Goals (SDGs) forth by the UN like: Goal 3 (Good Health and Well‐Being); Goal 8 (Decent Work and Economic Work); Goal 10 (Reduce Inequalities); and Goal 16 (Peace, Justice, and Strong Institutions) [18]. However, Construction sector is being a hazardous sector, especially in developing countries in absence of compliance to safety protocols. The scenario is more dangerous with three to four times higher accidents due to poor management and worker safety awareness [19]. Hydropower construction workers face even more risks due to civil structures, with a 0.69 risk probability of labor injuries and accidents annually [20, 21].

Occupational safety depends not only on the availability of protective measures but also on workers' knowledge and their translation of that knowledge into actual safe behaviors. While adequate knowledge is considered a prerequisite for safe practices, evidence suggests that knowledge alone does not always guarantee compliance with safety protocols. For example, a study conducted in Nigeria showed that, although a majority of the respondents had good knowledge (62%) of occupational hazard, nearly all (86%) reported poor practice of occupational safety measures [6]. Conversely, limited knowledge has been consistently associated with lower adherence to safety practices [1]. This highlights a complex interplay between what workers know and how they act in real workplace settings. Theoretical frameworks such as the Knowledge‐Attitude‐Practice (KAP) model and the Theory of Planned Behavior (TPB) provide useful lenses. The KAP model suggests that knowledge forms the basis for shaping attitudes, which in turn influence practice [22]. Similarly, TPB emphasizes that behavior is guided not only by knowledge and attitudes but also by subjective norms and perceived control. These frameworks allow a more nuanced understanding of why workers with comparable knowledge levels may differ in their occupational safety behaviors. Given these dynamics, it becomes crucial to study both knowledge and safety practices simultaneously, rather than in isolation. In Nepal, where numerous hydropower projects are under construction, workers are frequently exposed to occupational hazards. Despite the availability of safety guidelines, evidence suggests a gap between knowledge and practice [1]. According to the report of Nepal labor force survey 2017/18, 13.8% people are engage in construction sector [23]. Similarly, a study conducted on current situation of occupational health and safety by General Federation of Nepalese Trade Unions at 2012 showed that there was very low level of knowledge among worker in Kathmandu valley and almost nil outside the valley [24]. In the same way, study conducted among Nepalese construction worker on topic Knowledge and Understanding of Personal Protective Equipment Use among Laborer Population of the Nepalese Workforce at 2021 shows that 62% that is more than half of the participants had inadequate knowledge of OSH and the rest had an adequate level of knowledge 38%. Similarly, it has also found that 17% of participants did not use PPE and the prevalence of the occupational accidents within a year was 18% [1]. Another study conducted in Nepal by Shrestha SK at 2021 also showed that lack of proper knowledge of the hazards management, lack of understanding about the workplace safety rules and inadequate engagement of Safety were the main reason for hindering PPE utilization on workplace [12]. Therefore, recognizing the existing research gap, this study aimed to assess both occupational health knowledge and safety practices among construction workers at a hydropower construction site in Tanahun, Nepal, with the objective of identifying critical gaps and generating evidence to guide practical, context‐specific interventions for improving occupational health and safety in the construction sector.

## 2 Aim

This study aimed to assess the occupational health knowledge and safety practices of construction workers at a hydropower construction site in Tanahun, Nepal, with a focus on their knowledge of occupational health hazards, use of preventive measures, and related health problems. Specifically, it sought to examine the level of knowledge regarding occupational health hazards, the prevailing safety practices including the use of personal protective equipment, the common occupational health problems experienced, and the sociodemographic and work‐related factors associated with knowledge and practices. By addressing these aspects, the study fills a critical research gap, as limited evidence exists on occupational health and safety among hydropower construction workers in Nepal, and provides context‐specific evidence to guide policy‐makers, employers, and practitioners in designing targeted interventions to reduce work‐related injuries and promote safer working environments.

## 3 Methods

### 3.1 Study Design and Setting

A cross‐sectional study was conducted in Tanahun District, Nepal, among the construction workers who are engaged in the under‐constructing hydropower project. “Tanahun hydropower project” of Tanahun district is 140 megawatts medium‐sized hydropower plant, with a reservoir area of 7.26 sq.km, diversion tunnels, dam, gated spillway chute, intake, headrace and tailrace tunnels, powerhouse, and transmission line [25]. This was selected as the study site not only for accessibility but also because it reflects a typical mid‐scale hydropower construction environment in Nepal, employing a large and diverse workforce. As the project is in its early construction phase, involving dam, tunnel, powerhouse, access road, camp facilities, and transmission line works, it provides comprehensive exposure to occupational hazards, making it a representative and contextually appropriate setting to assess occupational health knowledge, safety practices, and related health problems.

### 3.2 Sample Size and Sampling Technique

The sample size was determined using Cochran's formula for estimating a proportion (*n *= z^2^pq/d^2^), with a 95% confidence interval, 5% margin of error, and an assumed prevalence (PPE use) of 25% based on a study conducted among construction workers in Gujarat, India [26], which was considered a reasonable proxy due to similar occupational and socioeconomic conditions in the construction sector. Substituting these values yielded an initial sample size of 288. To account for a potential 10% nonresponse rate, the sample size was increased to 316, which was taken as the final sample size. There were 1256 workers working in the construction site and a list of Construction workers was obtained from the office of Tanahun Hydropower Project. From this list, participants were selected using simple random sampling through a lottery method, where each worker's name was assigned a unique number and randomly drawn until the required sample size was reached.

### 3.3 Data Collection

A semi‐structure questionnaire was used in interviews to gather data on respondents' knowledge and practice. The data was collected from the October 2023 and November 2023. The interview was conducted in the private setting by the well ‐trained public health professional after the written informed consent from the study participants. The participants were informed on the objective of the study, rights to withdraw interview or skip the particular question, and voluntary participation. The questionnaire comprised of five sections. In the first three sections, questions related to demographics, occupation and knowledge were included whereas the rest of two sections include preventive practices, and occupational illness. The average interview time was 15–20 min for each participant.

The tools for the study were adapted from the previous studies and developed through the extensive literature review in English and then translated into Nepali language prior the pre‐testing. The forward and backward translation of the tools was conducted in consultation with subject experts. Before actual use, the tool was pre‐tested on 10% of construction workers working in electricity transmission line construction sector to ensure the face validity of tools and clarity. Cronbach's alpha was used to assess the tool's internal consistency; the result was 0.82 indicating good reliability. Additionally, test–retest reliability was performed on a subset of 20 participants over a 2‐week interval, showing satisfactory stability (intra‐class correlation coefficient = 0.76).

The study included construction workers employed at the Tanahun Hydropower Project who were present during the period of data collection and willingly consented to participate. Workers who could not adequately communicate in either Nepali or English, such as those speaking only Chinese or Hindi, were excluded to ensure proper understanding of the study tools and to maintain the accuracy and reliability of the data collected. All 316 participants responded completely, and no missing data or non‐responses were observed.

### 3.4 Study Variables

#### 3.4.1 Exploratory Variables

The socio‐demographic variables included in the first section. There were seven variables in this section comprising the participant's age, ethnicity based on the Health Management Information System's ethnic codes [27], education, which refers to the highest academic grade the participant has completed, family type, marital status, and living arrangements, which refers to whether the participant lives alone, with family, or with someone else.

The study also included 13 health problem related variables like experience of musculoskeletal problems, skin problems, head injuries, leg injuries, respiratory problems and Occupational factors like working experience, working hours, type of work to analyze the knowledge and safety practices of worker [28, 29].

#### 3.4.2 Outcome Variables

The study assessed two main outcome variables: knowledge and preventive practices related to occupational health hazards. The structured questionnaire consisted of knowledge and practice related sections. The knowledge section included 10 items on knowledge, covering areas such as sources of information, types of hazards, examples of physical, chemical, biological, and mechanical hazards, health problems due to exposure, and causes of accidents. The practice related section contained 18 items on preventive practices, focusing on aspects such as availability and use of PPE, frequency of use, and types of PPE utilized. Each item was scored as 1 for correct/positive responses and 0 for incorrect/negative responses. The total score for the multiple response question was calculated and then converted to a score out of 1. For example, if the multiple response question had 4 answers and the participant answered two options out of four answers, then the total score for it will be 2 which will be converted to 0.5 out of 1 which will be added later on to calculate total knowledge score of the participant. Composite scores were then calculated by summing responses within each section, yielding total knowledge and practice scores. The Kolmogorov Smirnov test was performed to test the normality of data and mean was identified as data was found with normal distribution. The mean knowledge score was 5.31, and the mean practice score was 11.25. Participants scoring below the mean were classified as having inadequate knowledge or poor practice, whereas those scoring at or above the mean were considered to have adequate knowledge and good practice [29].

### 3.5 Data Analysis Procedure

Collected data were entered in Excel using built‐in automated validation criteria to minimize entry errors. Then coding, classification and cleaning of data has been done manually. Entered data were analyzed through SPSS version 20. A total of 10% of the randomly selected data were manually rechecked for accuracy in data entry and any discrepancies identified were corrected through verification with the source data. Then, all data were exported to Statistical Package for the Social Sciences V.20 for the statistical analysis. The data were summarized in terms of frequency, percentage, mean, and standard deviation. Two separate binary logistic regression models were developed: one (M1) with knowledge of occupational health hazards (adequate vs. inadequate) as the dependent variable, and another (M2) with preventive safety practices (good vs. poor) as the dependent variable. Model included independent variables such as age, education, religion, duration of over time, training etc. Running separate models allowed a clearer understanding of factors uniquely associated with knowledge and with practice, rather than combining outcomes, which could have introduced collinearity and obscured distinct relationships. The Pseudo R^2^ was found 0.36 and 0.39 respectively for first (M1) and second (M2) model. The area under ROC curve were 0.867 and 0.872 respectively for both the model. These models were also compared with the null model and *p*‐value was found significant suggesting that the fitted model is better than null. Variables with *p*‐value < 0.25 in bi‐variate analysis were included in the multivariate analysis to calculate adjusted odds ratio. Variance Inflation Factor (VIF) less than 2 is used as an indication of no multicollinearity was detected. Variables with *p*‐value < 0.05 were considered statistically significant.

### 3.6 Ethical Statement

All methods of this study were carried out in accordance with the Declaration of Helsinki on ethical principles for medical research involving human participants. Ethical approval was obtained from the Institutional Review Committee (IRC) of Manmohan Memorial Institute of Health Sciences (MMIHS/IRC/080/065), and formal permission was obtained from Tanahun Hydropower Limited. Before data collection, all participants were informed in detail about the study objectives, procedures, voluntary participation, and their right to withdraw at any time, and written informed consent was obtained.

To ensure confidentiality, participants' personal identifiers were not recorded on the questionnaire. Data were coded using unique participant ID numbers, and all paper‐based forms were securely stored and accessible only to the research team. Electronic data were stored on password‐protected computers, with access restricted to authorized personnel. All reports and publications presented aggregated data, ensuring that no individual participant could be identified.

## 4 Results

The study involved 316 male participants mean aged 29.25 years (SD: ± 6.48), with Janajati being the most ethnic group. Majority of respondents was Hindu followed by Buddhist and Christian. Over half had completed primary level education followed by Secondary level and literate. In terms related to family type, more than two‐third of the respondents belonged to a joint family. The majority were married (Table 1).

**Table 1:** Socio‐demographic characteristics of the respondents.

Socio‐demographic characteristics	Category	Frequency (*n* = 316)	Percentage (%)
Age in years (Mean: 29.25, SD: ± 6.48)	< 20	9(2.8)	2.8
	20–29	173(54.7)	54.7
	30–39	114(36.1)	36.1
	> 40	20(6.3)	6.3
Sex	Male	316(100.0)	100.0
Ethnicity	Janjati	130(41.1)	41.1
	Chhetri	104(32.9)	32.9
	Madhesi	32(10.1)	10.1
	Dalit	26(8.2)	8.2
	Brahmin	24(7.6)	7.6
Religion	Hindu	265(83.9)	83.9
	Buddhist	38(12)	12.0
	Christian	13(4.1)	4.1
Education	Primary level	173(54.7)	54.7
	Secondary level	100(31.6)	31.6
	Literate	43(13.6)	13.6
Family type	Joint	217(68.7)	68.7
	Nuclear	81(25.6)	25.6
	Extended	18(5.7)	5.7
Marital status	Married	265(83.9)	83.9
	Unmarried	51(16.1)	16.1

The mean knowledge score was 5.31 (SD: 1.243), and the mean practice score was 11.25 (SD: 2.421). Participants scoring below the mean were classified as having inadequate knowledge or poor practice, whereas those scoring at or above the mean were considered to have adequate knowledge and good practice. Although all of them had heard about occupational health hazards, Majority of respondents had adequate knowledge and remaining had inadequate knowledge. Similarly, in terms of practice, Majority of respondents had good safety practice while slightly more than one‐third had poor practice (Table 2).

**Table 2:** Level of Knowledge of occupational health hazards and safety practices.

Knowledge and practice related variables	Category	Frequency (*n* = 316)	Percentage (%)
Knowledge of occupational health hazards of respondents	Adequate Knowledge	204	64.6
	Inadequate knowledge	112	35.4
Practice of respondents	Good practice	203	64.2
	Poor Practice	113	35.8

The study showed that majority encountered electric shock followed by wounds or cuts from sharp objects, head injuries, and endured hand and leg injuries. Similarly, out of total respondents, less than one‐third had faced skin‐related problems followed by Musculoskeletal Disorder, respiratory‐related problems, and ENT problems (Table 3).

**Table 3:** Prevalence of health problems among the respondents.

Health problems related variables	Category	Frequency (*n* = 316)	Percentage (%)
Occupational injuries			
Experience electric shock	Yes	46	14.6
Experience wound/cuts from sharp object	Yes	44	13.9
Experience Head Injuries	Yes	44	13.9
Experience Hand And legs injuries	Yes	43	13.6
Occupational Illness			
Experience skin related disease	Yes	88	27.8
Experience musculoskeletal disorder	Yes	68	21.5
Experience respiratory related problems	Yes	7	2.2
Experience ENT problems	Yes	14	4.4

*Note:* The percentage represent point prevalence.

The results of multivariate logistic regression reveals that adequate knowledge was 4.73 times (AOR = 4.73, 95% CI, 1.78– 12.41) more likely among the participants with completion of secondary level of education as compared to those with literate and participants with longer working hours were 2.95 times (AOR = 2.95, 95% CI, 1.28–6.81) more likely to have adequate level of knowledge than with shorter working hours In the same way, participants without experienced of musculoskeletal disorder were 9.59 times (AOR = 9.59, 95% CI, 3.49–26.35) more likely to be knowledgeable than with experience of it (Table 4).

**Table 4:** Predictors of knowledge of health hazards among respondents.

Variables	COR	95%CI	p value	AOR	95%CI	*p* value
Education						
Literate	Ref			Ref		
Primary	7.89	3.54–17.51	0.051	5.25	1.93–11.78	0.061
Secondary	13.39	5.59–32.09	0.021	4.73	1.78–12.41	**0.002** *
Overtime						
≤ 2	Ref			Ref		
> 2	2.32	1.32–4.08	0.004	2.95	1.28–6.81	**0.011** *
Experience injuries						
Yes	Ref			Ref		
No	3.21	1.31–7.48	0.041	2.14	0.75–6.08	0.153
Experience musculoskeletal disorder						
Yes	Ref			Ref		
No	4.65	2.21–9.81	0.001	9.59	3.49–26.35	**0.001** *

*Note:* Bold values represent the statistical association.

* Significance at *p* < 0.05.

The multivariate logistic regression showed that participants with higher level of education, like primary and secondary, were 9.59 (AOR = 9.59,95%, CI,3.33–27.62) and 4.75 (AOR = 4.75,95%, CI,1.24–18.22) times more likely to have good safety practice respectively than those who were literate only. Similarly, married participants were 16.41 (AOR = 16.41,95%, CI, 4.27–62.99) times more likely to follow good safety practices than unmarried participants and participants who work shorter duration were 1.86 (AOR = 1.86, 95% CI, 1.63–3.41) times more to pay attention to good safety practices than participants working longer duration. Additionally, participants without Musculoskeletal problems were 8.4 (AOR = 8.4, 95% CI, 1.73–40.74) times more adopt to undertake good safety practice than with musculoskeletal problems (Table 5).

**Table 5:** Predictors of good safety practice among the respondents.

Variables	COR	95%CI	p‐value	AOR	95%CI	*p* value
Religion						
Hindu	Ref			Ref		
Non‐ Hindu	2.28	1.12–4.64	0.024	1.51	0.54–4.24	0.432
Education						
Literate	Ref			Ref		
Primary	0.52	0.23–1.14	0.102	9.59	3.33–27.62	**0.001** *
Secondary	10.84	5.918–19.92	0.001	4.75	1.24–18.22	**0.023** *
Marital status						
Married	12.75	5.91–27.53	0.001	16.41	4.27–62.99	**0.001** *
Unmarried	Ref			Ref		
Over time						
≤ 2	2.29	1.38–3.78	0.001	1.86	1.63–3.41	**0.037** *
> 2	Ref			Ref		
Experience electric shock						
Yes	2.22	1.18–4.17	0.013	0.62	0.21–1.85	0.391
No	Ref			Ref		
Experience musculoskeletal problems						
Yes	Ref			Ref		
No	17.271	5.285–56.44	0.001	8.4	1.73–40.74	**0.008** *
Receive training						
Yes	2.407	1.112–5.211	0.23	2.25	0.57–8.85	0.246
No	Ref			Ref		

*Note:* Bold values represent the statistical association.

* Significance at *p* < 0.05.

## 5 Discussion

This study revealed that nearly two‐thirds (64.6%) of respondents had adequate knowledge of occupational health hazards. This is substantially higher than the 33.3% reported in a previous study among the general labor workforce in Nepal [1]. The disparity may be explained by the presence of safety officers on hydropower construction sites, who likely reinforce awareness and compliance with safety standards. Education emerged as a strong predictor of knowledge, with respondents having higher education levels showing significantly greater awareness (AOR = 4.73, 95% CI: 1.78–12.41). This finding is consistent with research in Ethiopia (AOR = 20.03, 95% CI: 16.30–23.75) [30], highlighting the critical role of education in shaping occupational health literacy. However, studies from Sri Lanka and the Philippines did not find such an association [31, 32], possibly due to contextual differences such as accessibility to education, workplace culture, and health service availability.

Longer working hours were also positively associated with knowledge in this study, echoing findings from the Philippines [32]. Increased exposure to workplace hazards may explain this pattern, as workers gain experiential knowledge over time. Notably, almost all participants (97.8%) reported working overtime, which aligns with findings from Pokhara [29]. Nevertheless, this widespread practice contravenes Nepal's Labor Act, 2074, which restricts working hours to eight per day [16], underscoring a gap between policy and practice.

Regarding safety practices, 64.2% of respondents demonstrated good preventive behaviors, a considerably higher proportion than the 36.2% reported among construction workers in Pokhara [29]. This improvement may reflect stricter enforcement of safety protocols and incentive mechanisms in the hydropower sector. Education again played a decisive role, with those attaining higher education more likely to engage in safe practices (AOR = 4.75, 95% CI: 1.24–18.22). This aligns with findings from Ethiopia (AOR = 4.3, 95% CI: 2.4–7.8) [33] and other contexts including Nigeria and the Philippines [29, 32, 34]. Conversely, a study among fabrication workers in Kathmandu found no association between education and practice [AOR = 0.46, CI = 0.005–47.50) [35], suggesting that sector‐specific conditions, such as PPE availability and workplace culture, may moderate this relationship.

Work hours also influenced practice, with shorter working hours linked to better compliance. Studies from Malaysia and South Florida support this, showing that extended workdays foster fatigue, impair concentration, and discourage PPE use [36, 37]. Moreover, workers without musculoskeletal disorders (MSDs) were more likely to report good practices, consistent with evidence from Saudi Arabia [38] suggesting that adherence to safety measures reduces MSD risk. Marital status was also associated with safety practices, with married workers more compliant; however, studies from Turkey and the Philippines did not support this association [32, 39], likely reflecting cultural and socioeconomic variations. Interestingly, training did not significantly predict practice in this study (AOR = 2.25, 95% CI: 0.57–8.85), in contrast to studies in Ethiopia (AOR = 12.8, 95% CI = 6.3–26.1) [33] and another study in same country (AOR = 3.73; 95% CI: 2.33–5.98) [40], where lack of training was a major predictor of poor practices. Some variables in this study showed differences between crude and adjusted regression results that can influence the interpretation of findings by indicating the presence and effect of potential confounding variables. Crude estimates show the unadjusted association between an exposure and an outcome, while adjusted estimates account for other variables that might influence this relationship. When an association observed in the crude analysis becomes weaker or nonsignificant after adjustment, it suggests that the initial relationship was partly or fully explained by confounding factors. Conversely, if the association remains strong or becomes more pronounced after adjustment, it indicates an independent effect of that variable after controlling for confounders. In this study, some crude associations such as those between religion and safety practice lost statistical significance after adjustment, suggesting confounding by variables like education and marital status. On the other hand, the associations of education and musculoskeletal disorders with knowledge and safety practice remained significant even after adjustment, demonstrating their independent influence.

The study also documented the prevalence of occupational injuries and illnesses. Electric shocks (15%), wounds/cuts (14%), and injuries to the head, hand, and leg (14% each) were most common, primarily caused by slipping, tripping, falling, and hand tools. These findings resemble those from hydropower workers in Ethiopia [41] but differ from a Nepalese workforce study where lifting and carrying objects predominated [1], underscoring the influence of specific work environments on injury patterns. Similarly, skin infections (27.8%), musculoskeletal problems (21.5%), respiratory issues (2.2%), and ENT problems (4.4%) were prevalent, aligning with findings among underground construction workers in Nepal [42]. Compared to the Kashmir Valley [11], prevalence rates were lower, likely due to broader PPE availability in the current study.

This study underscores the importance of education, working conditions and duration of work in shaping workers' knowledge and practices regarding occupational health and safety. The high levels of knowledge and safety practices observed among hydropower construction workers highlight the potential impact of structured safety policies, dedicated safety officers, and consistent PPE use. However, challenges remain, particularly regarding long working hours and inadequate training, and policy enforcement. Strengthening occupational health education, ensuring compliance with labor regulations, and institutionalizing comprehensive training programs are essential for minimizing workplace hazards and improving worker health outcomes. These findings carry significant implications for policymakers, employers, and occupational health practitioners striving to safeguard the well‐being of construction workers in Nepal and comparable settings.

### 5.1 Strength and Limitation of the Study

This study has several strengths. It is among the few studies to examine occupational health and safety within Nepal's hydropower construction sector, a high‐risk yet underexplored industry. By focusing on this sector, the study contributes to filling a critical knowledge gap in occupational health research in Nepal and provides evidence that can inform both policy and practice. A further strength lies in its assessment of multiple dimensions of occupational health and safety, including workers' knowledge, practices, educational attainment, working hours, and health‐related conditions. This multidimensional approach enhances the study's relevance for workplace interventions and policymaking in comparable contexts.

Nevertheless, some limitations should be acknowledged. First, the cross‐sectional design of the study limits the ability to establish causal relationships between occupational exposures and health outcomes. Second, the reliance on self‐reported data introduces the potential for recall bias and social desirability bias, which may affect the accuracy of reported practices and health experiences. Third, the use of mean‐based cut‐off points to classify knowledge and practices may constrain external validity and might also introduce misclassification bias. For example: when we use the mean as a cut‐off, participants whose scores are near the mean might be arbitrarily classified into different groups. Small differences in scores around the mean may not represent meaningful differences in the construct being measured, leading to misclassification. In addition, the absence of qualitative data restricted the exploration of workers' lived experiences, attitudes, and contextual challenges related to occupational health and safety. The exclusion of female also limits the generalizability of findings. Finally, while the focus on hydropower construction provides valuable sector‐specific insights, it also restricts the generalizability of findings to other segments of the construction industry or different occupational groups in Nepal.

### 5.2 Study Implication

The findings of this study carry important implications at multiple levels.

At the policy level, the results underscore the need for stronger enforcement of existing labor regulations and the development of sector‐specific occupational health and safety policies for hydropower construction. Policies should encourage the integration of engineering controls such as improved ventilation systems to reduce dust and fumes, ergonomic redesign of tools and equipment to minimize musculoskeletal strain, and adequate drainage and protective materials to prevent skin exposure to cement and chemicals. Addressing worker safety in this industry is not only a matter of labor rights but also aligns with Nepal's broader commitments to achieving the SDGs, particularly those related to decent work, health, and sustainable economic growth.

At the practice level, for employers and industry stakeholders, the study highlights the importance of systematic worker training, health education, and the provision of adequate protective measures. Organizational controls such as task rotation to reduce repetitive strain, enforcement of rest breaks, monitoring of noise and dust levels, and establishment of regular health checkups can help prevent skin, musculoskeletal, ENT, and respiratory disorders. Identifying high‐risk groups within the workforce points to the need for targeted workplace interventions that address disparities in education, health status, and working hours. Such engineering and organizational measures can directly improve safety practices and reduce occupational health risks.

At the research level, the study points to the need for further investigation into occupational health and safety in Nepal's construction sector. Future research should adopt longitudinal designs to establish causal pathways between specific workplace exposures (e.g., noise, dust, repetitive tasks, and chemical contact) and health outcomes. Incorporating qualitative methods would also provide deeper insights into workers' perceptions, experiences, and contextual barriers, complementing the quantitative findings and enriching evidence for policy and practice.

### 5.3 Recommendations

Based on the findings, it is recommended that hydropower companies, in collaboration with the Department of Labor, prioritize quarterly safety training for high‐risk groups such as workers with musculoskeletal problems, long working hours, and low educational attainment, focusing on hazard prevention and the proper use of personal protective equipment. The government should strengthen the enforcement of labor regulations by conducting routine inspections, at least twice a year, to ensure compliance with limits on working hours. At the workplace level, companies should carry out monthly site inspections to promptly address hazards, ensure functional first‐aid facilities, and organize participatory safety meetings to incorporate worker feedback into safety planning. Finally, both government and industry stakeholders should establish a joint monitoring mechanism to review occupational health and safety programs annually, allowing continuous improvement and sustained reduction of workplace accidents and health risks. For future research, qualitative or mixed‐methods approaches are recommended to explore workers' perceptions of occupational risks, barriers to compliance, and the contextual factors influencing health and safety behaviors. Such studies would complement the quantitative evidence from this study and help design more feasible and context‐specific interventions in Nepal's construction sector.

## 6 Conclusions

This study highlights the critical importance of occupational health knowledge and safety practices among workers in Nepal's hydropower construction sector, a field characterized by high exposure to physical and environmental hazards. The findings contribute valuable evidence on how workers' educational level, working conditions, and health status influence their safety behaviors, offering insights that can inform more effective workplace interventions and policy actions in Nepal's growing infrastructure industry. While the study demonstrates the relevance of knowledge and preventive practices in mitigating occupational health risks, its interpretation should be viewed within certain limitations. The cross‐sectional design restricts causal inference, the male‐only sample limits generalizability to female workers, and reliance on self‐reported data may introduce recall or social desirability bias. Future research should adopt longitudinal designs to clarify causal relationships between workplace exposures and health outcomes, include female construction workers to understand gender‐specific risks, and incorporate qualitative or mixed‐methods approaches to explore workers' perceptions of safety culture and barriers to compliance. Collectively, these efforts can advance a more comprehensive understanding of occupational health and safety in Nepal's hydropower sector and support evidence‐based improvements in worker protection.

## Author Contributions

Authors contributed in the following ways: study design (Khadga Bahadur Shrestha, Swastika Gairhe, Amrit Bist, Mamata Rawal) implementation (Swastika Gairhe), analysis (Khadga Bahadur Shrestha, Swastika Gairhe, Mamata Rawal, Amrit Bist) and writing (Swastika Gairhe, Amrit Bist). All authors reviewed and approved the final version of the manuscript.

## Disclosure

The lead author Amrit Bist affirms that this manuscript is an honest, accurate, and transparent account of the study being reported; that no important aspects of the study have been omitted; and that any discrepancies from the study as planned (and, if relevant, registered) have been explained.

## Conflicts of Interest

The authors declare no conflicts of interest.

## Data Availability

The authors are committed to providing access to the raw data and supporting materials upon reasonable request, ensuring that the research is open and verifiable.
